# Addressing bottlenecks in Lassa fever treatment: overcoming the ribavirin parenteral formulation challenge

**DOI:** 10.1186/s41182-024-00609-0

**Published:** 2024-07-08

**Authors:** Qudus Olajide Lawal, Joseph Okoeguale, Sebastine Oseghae Oiwoh, ThankGod Akhigbe, Reuben Agbons Eifediyi, Sylvanus Akhalufo Okogbenin

**Affiliations:** 1 Department of Obstetrics and Gynaecology, Irrua Specialist Teaching Hospital, Irrua, 310114 Edo State Nigeria; 2 Department of Internal Medicine, Irrua Specialist Teaching Hospital, Irrua, 310114 Edo State Nigeria

**Keywords:** Ribavirin, Lassa fever, Antiviral therapy, Dose formulation, Ampoule

## Abstract

Ribavirin ampoule formulation remains a major challenge in managing Lassa fever disease. Lassa fever is an endemic viral hemorrhagic fever in the West Africa subregion, which has high-dose ribavirin as the standard of care. The high-dose therapy required makes the 200 mg/ml ampoule dosing of ribavirin a daunting task to administer, especially during disease outbreaks. This commentary highlights the challenges and makes a passionate call for vial dosage adjustment to fit the high-dose requirement of Lassa fever disease.

Ever since it was first patented in 1972 [1], ribavirin has found various important roles in the management of broad-spectrum RNA viral diseases and some DNA viral diseases [2, 3]. It was first used for the management of Respiratory Syncytial Virus (RSV) in children in aerosol form [3], and the oral form remains an essential component of the management of the Hepatitis C virus (HCV) [4]. However, it is in the management of Lassa fever that its intravenous formulation has made the most remarkable impact where it remains the standard of care [5, 6].

Lassa fever infection is an endemic viral hemorrhagic fever in the West Africa subregion caused by a zoonotic infection transmitted by a multimammate rat**,** *Mastomys natalensis* [7]. The disease is highly contagious and associated with a high case fatality rate, especially during outbreaks, with an estimated 100,000–3,000,000 new infections and approximately 5000 deaths per annum [6, 8]. Ribavirin, especially, when started in the early course of the disease, improves patient outcome [5, 9].

Ribavirin is a broad-spectrum synthetic nucleoside analogue with multiple proposed mechanisms of action including viral lethal mutagenesis [2]. In the management of Lassa fever, there are two regimens for the administration of Adult Ribavirin, both regimens involve the administration of intravenous Ribavirin over a 10-day period [5, 10]. In the McCormick, intravenous Ribavirin is administered at a loading dose of 33 mg/kg (maximum 2.64 g), followed by 16 mg/kg (maximum 1.28 g) 6 h for 4 days, followed by 8 mg (maximum 0.64 g) 8 h for another 6 days [5]. The Irrua regimen uses daily dosing of 100 mg/kg (maximum 7 g), followed by 25 mg/kg daily days 2–7, and followed by 12.5 mg from days 8–10 [10]. In pregnancy, a modified McCormick regimen is used with a loading dose of 100 g/kg divided doses in place of 33 mg/kg stat dose [10].

Available intravenous Ribavirin is formulated as 200 mg /ml in a 1 ml ampoule which makes administration of the medication cumbersome. For an adult loading dose, depending on the regimen, 10–23 ampoules of Ribavirin will need to be broken (Fig. 1). Several ampoules will need to be broken in the 10-day course of treatment, translating to millions of ampoules in the endemic West Africa sub-region. This becomes more problematic during case surges when up to 189 patients can be admitted in a quarter [8].

**Fig. 1:**
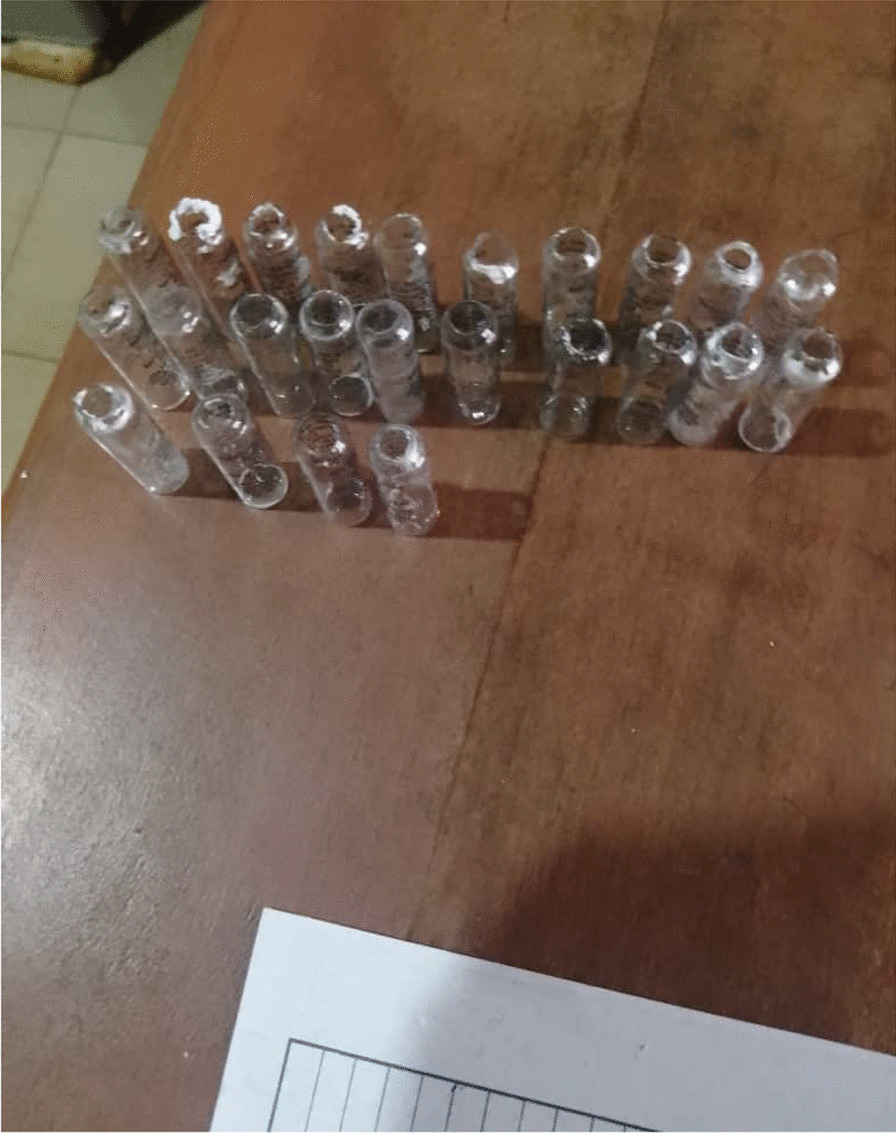
Ampoules of Ribavirin administered to a patient during Lassa fever outbreak

The logistics of breaking multiple ampoules, even with the single-dosing Irrua regimen, may result in missing doses, with dire consequences to disease outcomes. It also results in inefficient time management, especially during disease surges. The opportunity cost of diverting resources away from other critical healthcare tasks is enormous.

Given its central role in the management of Lassa fever, it is high time high-concentration Ribavirin formulations or high-volume vials are produced. This innovative approach is feasible and the implication of implementing this strategy will help improve the efficiency of management of Lassa fever.

Stakeholders, including healthcare providers, pharmaceutical companies, and policymakers need to come together to address this seemingly minor problem with a huge impact in optimizing treatment processes to enhance healthcare delivery.

## Data Availability

Not applicable.
